# Valorizing alum sludge waste augmented ferrite as a sustainable magnetic pathway for treating Indigo carmine effluent

**DOI:** 10.1038/s41598-025-13300-z

**Published:** 2025-08-12

**Authors:** Ahmed H. Mangood, Eman Sh. Salama, Ibrahim E. T. El-Sayed, Mai K. Fouad, Maha A. Tony

**Affiliations:** 1 Chemistry Department, Faculty of Science, Menoufia University, Shebin El-Kom, Egypt; 2 Advanced Materials/Solar Energy and Environmental Sustainability (AMSEES) Laboratory, Faculty of Engineering, Menoufia University, Shebin El-Kom, Egypt; 3 Chemical Engineering Department, Faculty of Engineering, Cairo University, Giza, Egypt; 4 Planning and Construction of Smart Cities Program, Faculty of Engineering, Menoufia National University, Menoufia, 32651 Egypt

**Keywords:** Sorption, Indigo carmine dye, Alum sludge, Ferrites, Adsorption isotherms, Kinetics

## Abstract

One of the guiding sustainability principles is centered on mitigating the waste streams through the industrial ecology manner. On this regard, this research examines the conversion of dewatered alum sludge (AS) waste derived from water-works plants to be and innovative, magnetic and inexpensive nanoadsorbent. Alum sludge (AS) is calcined at 400 °C and mixed with zinc ferrite (F-Zn) that is prepared by simple co-precipitation route and signified with its high chemical stability, harmfulness as well as good magnetic properties that makes them a candidate as reusable adsorbent. The composite is mixed at varied proportions and labeled as AS400 (F-Zn/AS400 (1:1), F-Zn/AS400 (2:1) and F-Zn/AS400 (1:2). Such materials are thereby checked for their composition, structure and physical–chemical characterized through X-ray diffraction, Fourier transform infrared spectroscopy, transmission electron microscopy, scanning electron microscopy, Energy dispersive X-ray Spectroscopy, vibrating sample magnetometer, and Brunauer–Emmett–Teller. Then, the composites are applied for anionic dye so-called Indigo carmine (IC) adsorption through a comparative manner. The operating parameters are examined and the experimental results revealed that the isotherm time for all adsorbents is corresponding to 2 h using 0.5 g/L of the applied materials dose. Also, the aqueous medium pH is checked and the point of zero charge is evaluated and confirmed the IC removal was successful in an acidic medium (pH 2.0). The temperature influence verified the process is exothermic in nature. Kinetic modeling is evaluated and the results are well fitted with the pseudo-second order model. Various isotherm models are applied and the data is fitting the Langmuir isotherm model. The presence of ferrite improves the AS400 capacity from to 12.57 to 29.42 mg/g.

## Introduction

Globally, the water demand is in incredible increase due to modernization, industrialization and population increase. According to the United Nations World Water Development Report released in 2018 and the World Water Assessment Program, water demand will double by 2050 and a result excess than half of the world wide’s population will be at risk of water shortages^1^. Thereby, in recent decades, the rapid rise of industrial activities is leading to an increase in water pollution that eventually affects humans, animals and also the whole ecosystem. One of the most common wastewater discharges is wastewater contaminated with dyes. More than 700,000 metric tons of dyes are produced annually and applied for different applications^2^. The result is massive amounts with unlimited quantities of wastewater discharge contaminated with such synthetic dyes that are generated from varied industries including food, paper, fabric, rubber, plastics and electroplating industries^3^. For instance, textile industry that is signified as one of the water-intensive industries, for the object of fabricating 8000 kg of fabric in textile mills, average amount of about 1.6 million liters of water per day is used^4^. Among such used dyes, indigo carmine (IC), which also known as acid blue is widely applied. Its importance is gained since it is applied in manufacturing various products in the fields of printing, cosmetics, biological staining, dermatological and antibiotic agents, and poultry feed additives. Such dye has a strong irritating, recalcitrant, and poisonous effects on mammalian cells in addition to its high ability to color aqueous solutions^5^. Thus, its high hazardous effects on environment and public health, such dye-containing solutions treatment are a must^3^. Such job is the role of academia and industrial sector responsibility.

Dyes are highly toxic substances and traditional treatment methods are insufficient due to their drawbacks including long treatment time, costive, the presence of secondary pollutants and final sludge formation. In this regard, standard physical treatments such as filtration, settling, and other techniques, is not recommended as these approaches are ineffective for treating this type of contaminants. Thus, using conventional techniques in treatment is not recommended. Searching for an ecologically sustainable efficient treatment technique is still gaining the researchers’ interest to reach to safe treatment and nontoxic disposal. Various alternative techniques, such as membrane separation, reverse osmosis, coagulation, oxidative remediation, and adsorption, among others stand out for their ability to remove dyes from an aqueous medium^6^.

Dye adsorption from polluted is gained a considerable attention due to its significance treatment specially when it is conducted under environmental ecology materials. Also, adsorption is categorized as one of the most efficient methods for treating wastewater because it possess some merits including its low cost, simplicity in application, high efficiency, selectivity and sensitivity to toxic pollutants, reasonable treatment time and lack of sludge development^7,8^. But, the use of adsorbent materials is costive that is signified as a drawback of the adsorption system. Also, the primary objectives of the sorbent materials is to increase the selectivity of the sorbent materials and minimal production costs, explore opportunities for regeneration and searching for a safe disposal of such sorbents are improving the overall sorption efficiency. Such group limitations could be overcome by the application of waste based materials as an ecologically sustainable approach. Hence, applying natural organic and inorganic materials as well as industrial waste streams to be a value-added adsorbent material is a research topic^9^.

Alum sludge is one of inescapable by-products generated from treating water in drinking water-works plants. Such sludge is released due to the addition of aluminum sulfate in the coagulation process and categorized as waste material. Direct disposal of this type of sludge into the environment causes a severe damage to the ecosystem as well as adverse environmental effects and thereby causing hazards includes damage to the aquatic life since it contains high levels of aluminum concentration. Such hazards include damage to the aquatic life^10^. But, on the other hand, recycling alum sludge is presenting positive effects on the environment and the economy. For instance, there are numerous advantages of using this waste as an adsorbent material including use of an easily accessible and inexpensive material for the removal of pollutants from contaminated water^10,11^ as well as reducing the waste streams. However, alum sludge separation from recovered aqueous medium remains a barrier, restricting its actual industrial applications, despite its effectiveness in water remediation.

Various methods have been suggested recently for creating innovative composites that are affordable and useful for the removal of dyes. Composite material is a suitable candidate since they possess the advantage of increasing the treatment efficiency by combining the merits of using two or more materials in a certain way compared to the pristine use of their individual use. Thus, shows tremendous potential and customizable characteristics^12^. Ferrite substances showed a potential benefits when augmented with alum sludge as a treatment system. Also, such materials are highly recommended in treatment due to their possibility of separation from aqueous media after utilization. Ferrites is magnetic materials and could be a nanoscale substances with a wide range of physiochemical and functional characteristics. Their high surface range-to-volume ratio and nanosized are further benefits, in addition to their high saturation magnetization^13^. There are four subgroups of magnetic ferrites that are commonly recognized: garnet, orthoferrite, hexagonal ferrite, and spinel ferrite. Previously, a common formulation for spinel-type ferrite NMs has been M-Fe_2_O_4_ (M = Mn, Fe, Co, Ni, and Cu), which has been applied extensively in wastewater treatment^13,14^. Depending on how the cations are distributed in the tetrahedral or octahedral site, spinel ferrites can be classified into three major categories. ZnFe_2_O_4_ is a typical spinel ferrite in which Fe^3+^ ions are found in octahedral [B] sites and Zn^2+^ ions are found in tetrahedral [A] sites^15^. F-Zn stands out among all metal ferrites due to its exceptionally high chemical stability, magnetic qualities, and lack of toxicity to living things, and environmental safety^8^. Also, it could be more common due to its inexpensive cost, environmental friendly, and it is abundant in nature^14^. Additionally, F-Zn could be prepared by various techniques such as sol–gel^16^, hydrothermal route^17^, co-precipitation method^18^ and solvothermal method^19^.

According to the authors’ knowledge the novel composite is not applied so-far or may be used in wastewater treatment. Herein, the current investigation is exploring the novelty of using F-Zn nanoparticle that is synthesized via co-precipitation route and thereby mixed in various proportions with alum sludge that is calcined at 400 °C (AS400) to attain three composites namely F-Zn/AS400 (1:1), F-Zn/AS400 (2:1) and F-Zn/AS400 (1:2). The crystalline structure, morphology, and magnetization characterization are investigated. Next, the pristine materials ferrite and AS400 and their composites were applied as adsorbent material for indigo carmine (IC) dye removal. Additionally, thermodynamic parameters are investigated and kinetic and isotherm models are applied to best fit the experimental.

## Experimental investigation

### Adsorbent materials

Initially, (F-Zn) nanoparticle is prepared by simple co-precipitation from the aqueous solutions of zinc chloride (ZnCl_2,_ 136.30 g/mol, ≥ 98.0%, Sigma-Aldrich) and ferric chloride (FeCl_3,_ 162.20 g/mol, 97.0%, Sigma-Aldrich )precursors in an alkaline medium. It is always assumed that the initial molar proportion [Zn]:[Fe] is in stoichiometric ration of 1:2. Thus, the stoichiometric amounts of 0.1 M ZnCl_2_ and 0.2 M of ferric chloride were added in 250 ml distilled water and kept at constant stirring. Afterwards, a solution of 3 mol/L of sodium hydroxide (NaOH, 40.00 g/mol, ≥ 99.0%, Sigma-Aldrich) then added gradually until a precipitate is attained and kept for 15 min under stirring at room temperature. The mixture is then heated for 2 h at 80 °C. then, the collected precipitate is successively washed with distilled water till the pH of the solution is become neutral. The collected ferrite powder material is then overnight dried at 80 °C to attain a dry powder. The produced material is then subjected for different characterization^20^.

In Southern Shebin El-Kom City, Menoufia governorate, Egypt, alum sludge (AS) is collected from the water-works plant that is signified as the greatest water-works plant in such city. In such plant the water is pumped from the River Nile reservoir. In the plant aluminum sulphate is applied as the primary coagulant and the result is alum sludge that is generated as a by-product that is collected for study from the underflow channel of the sedimentation tank. The collected sludge is transferred into the laboratory and stored in plastic containers according to the standard methods till use in the study. The contained water in the sludge is subsequently removed by gravity settling. Then, air-drying is applied to reduce the moisture content in the sludge into less than 10%. The resultant sludge cake is subsequently cleaned to remove impurities by washing. Subsequently, AS is dried in an oven overnight at 105 °C to eliminate any remaining water content. Thereafter, the resultant sludge is subjected for one hour in a ball mill to attain a fine powder. The resultant material is labeled as raw alum sludge (AS) sample. Subsequent, AS is calcined for two hours at 400 °C in an electrical furnace, and the sample is named AS400.

Afterwards, in order to prepare the composite materials, the prepared ferrite (F-Zn) is mixed with modified alum sludge (AS400) in different proportions (1:1), (2:1) and (1:2) and labeled as -Zn/AS400 (1:1), F-Zn/AS400 (2:1) and F-Zn/AS400 (1:2) that are then subjected for characterization prior it is applied as adsorbent material^21^.

### Dye-containing wastewater

Synthetic wastewater sample that is signified as a simulated textile industry discharge is prepared as a model pollutant. Indigo carmine (IC) (C_16_H_8_N_2_Na_2_O_8_S_2,_ 466.35g/mol, 85.0%, Sigma-Aldrich) that is categorized as one of the extremely available dyes in the textile industry is used as received without further purification. The dye molecular weight is 466.36 g/mol. Such dye possess an organic molecule with the chemical formula 5, 5-indigodisulfonic acid sodium salt (C_16_-H_8_N_2_Na_2_O_8_S_2_), with four aromatic and two sulphonated groups.

Primarily, a stock solution of 1000 mg/L is prepared whereas extra dilutions are done, when needed, according to the studying range from 5 to 40 mg/L. Afterwards, a volume of 20 mL of dye-containing solution was used in all the adsorption experiments and the system is subjected for sorption facility after adding varied adsorbent materials according to the experimental conditions.

### Adsorption test and analytical determination

20 mL of IC solution was placed into 100 mL Erlenmeyer flasks for dye adsorption test. Also, the effects of various adsorption parameters on the dye removal efficiency and adsorption capacity are evaluated after investigating the isotherm time that is assessed in the range of 5–1440 min. Such parameters is including the initial operating pH of the aqueous solution that is checked from the acidic to the alkaline range (pH 2.0–10.0), temperature influence that is evaluated from the range of room temperature to 60 °C, composite dose (0.5–4 g/L) and initial IC dye concentration (5–40 mg/L). Furthermore, each parameter is evaluated when the other parameters are kept at their constant levels. The initial operating pH of the solution is adjusted, when required into the required values through the use of diluted sulfuric acid (1:9) (H_2_SO_4_, 98.08 g/mol, 99.9%, Sigma-Aldrich) and/or 1 M NaOH solutions using digital pH meter (AD1030, Adwa instrument, Hungary).

UV–vis spectrophotometer (Model Unico UV-2100, USA) was used to measure the residual IC dye concentration after adsorption process in the aqueous solution. Consequently, the samples were withdrawn and assessed at the maximum wavelengths of 610 nm. Equations (1) and (2) are applied to calculate the adsorption capacity (${\text{q}}_{\text{e}}$) and removal yield, respectively.

1 $${\text{q}}_{{\text{e}}} = \left( {{\text{C}}_{{\text{i}}} - {\text{C}}_{{\text{e}}} } \right)\frac{{\text{V}}}{{\text{m}}}$$

2 $${\text{R }} = \frac{{\left( { C_{i} - C_{e} } \right)}}{{C_{i} }} \times 100\%$$

where the initial and residual IC dye concentrations in the aqueous solution are, respectively, C_i_ (mg/L) and C_e_ (mg/L). Additionally, the mass of the dry adsorbent is represented as m in (g) and the solution volume by V (L).

**Fig. 1:**
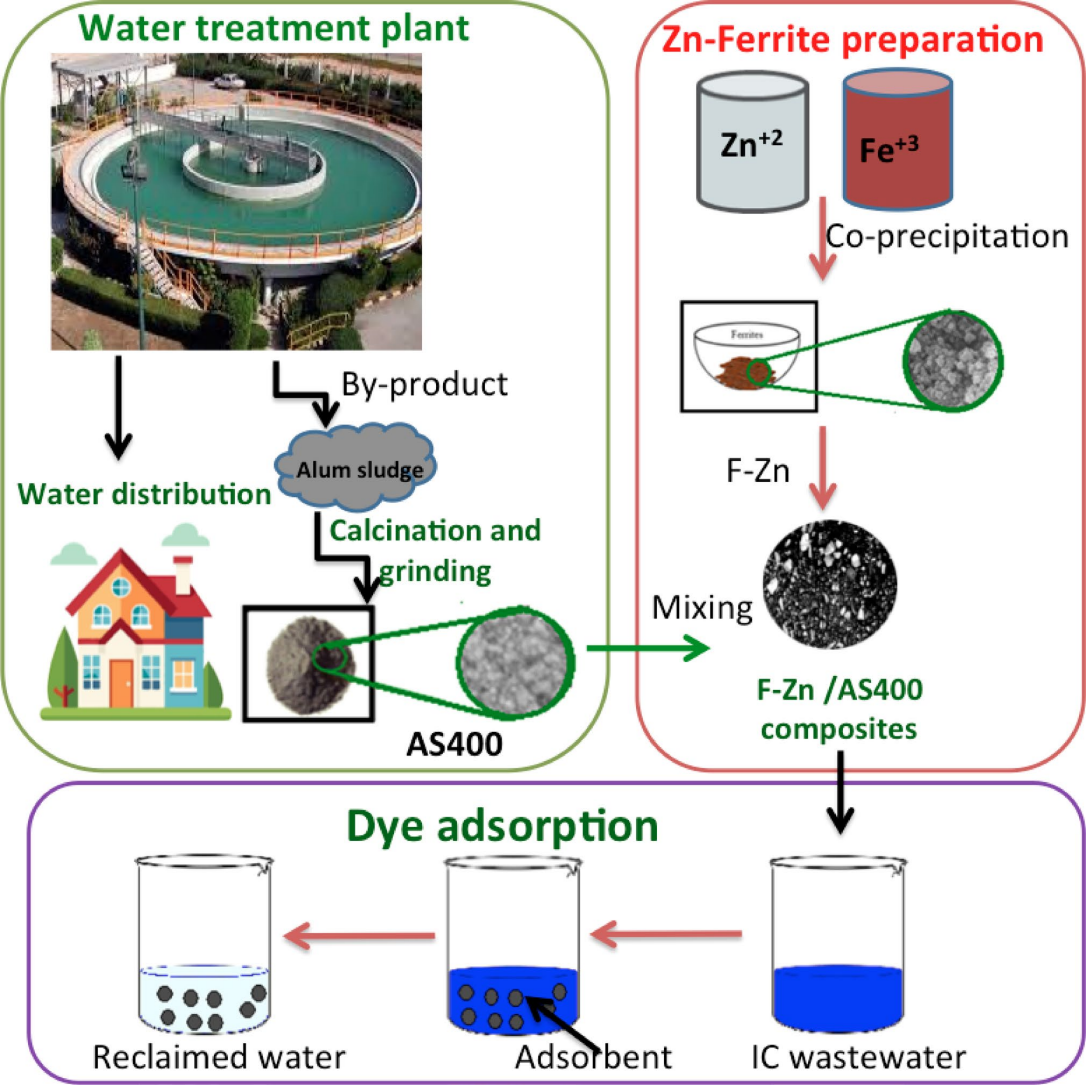
Graphical illustration of the experimental set-up.

The graphical scheme of experimental set-up illustration and reaction steps is exposed in Fig. 1.

### Point of zero charge (pHpzc)

In order to study the electrokinetic characteristics of a surface, or pH level at which the charge on the adsorbent surface approaches zero, pHPZC is calculated using the salt addition method. Using this approach, 25 mL of 0.01 M sodium chloride (NaCl, 58.44 g/mol, 99.0%, Sigma-Aldrich_)_ and 0.15 g of adsorbent were combined in 50 mL plastic tubes. A pH meter (C5010, Consort, Belgium) was used to adjust the pH to 2–12 using 0.1 M H_2_SO_4_ and 0.1 M NaOH. For twenty-four hours, the mixture was stirred in a shaker (Orbi shaker BT3000, Benchmark, USA). Plotting the difference between the initial and final pH against the initial pH allowed for the determination of the final pH.

### Characterization of the prepared adsorbents

X-ray diffraction (XRD) is signified as a useful technique for examining the crystalline and amorphous properties of the material being studied, as well as the phase structure of the generated samples. In this regard, the samples are examined using Cu-Kλ radiation at room temperature (λ = 1.54060 Å) operated at 40 kV and 30 mA utilizing X-ray powder diffractometry (XPERT-PRO diffractometer). Adsorption–desorption isotherm measurements of N_2_ at 80 K (BEL SORP MAX: Made in Japan) are used to determine the specific surface. The magnetic properties are also measured and evaluated through hand-made VSM DMS-880 instrument installed at Tanta University in Egypt, Physics Department of the Faculty of Science. In addition, Fourier transform infrared (FTIR) transmittance spectrum analysis are performed for all the prepared samples using Jasco FTIR-4100 spectrometer and conducted in the region of wavenumber of 400–4000 cm^−1^. Brunauer–Emmett–Teller (BET) surface is also assessed using Quantachrome operator. To examine the morphology of the prepared adsorbents a high-resolution Transmission Electron Microscope (TEM) analysis has been conducted (HR-TEM, Talos F200i-transmission electron microscope, Thermo Fisher Scientific Co., Eindhoven, and the Netherlands). Also, Scanning electron microscopy (SEM) analyses are perofrmed at an accelerating voltage of 25–30 kV model Philips XL 30 (Eindhoven, Netherlands).

## Results and discussions

### Characterization of the prepared adsorbents

#### XRD analysis

**Fig. 2:**
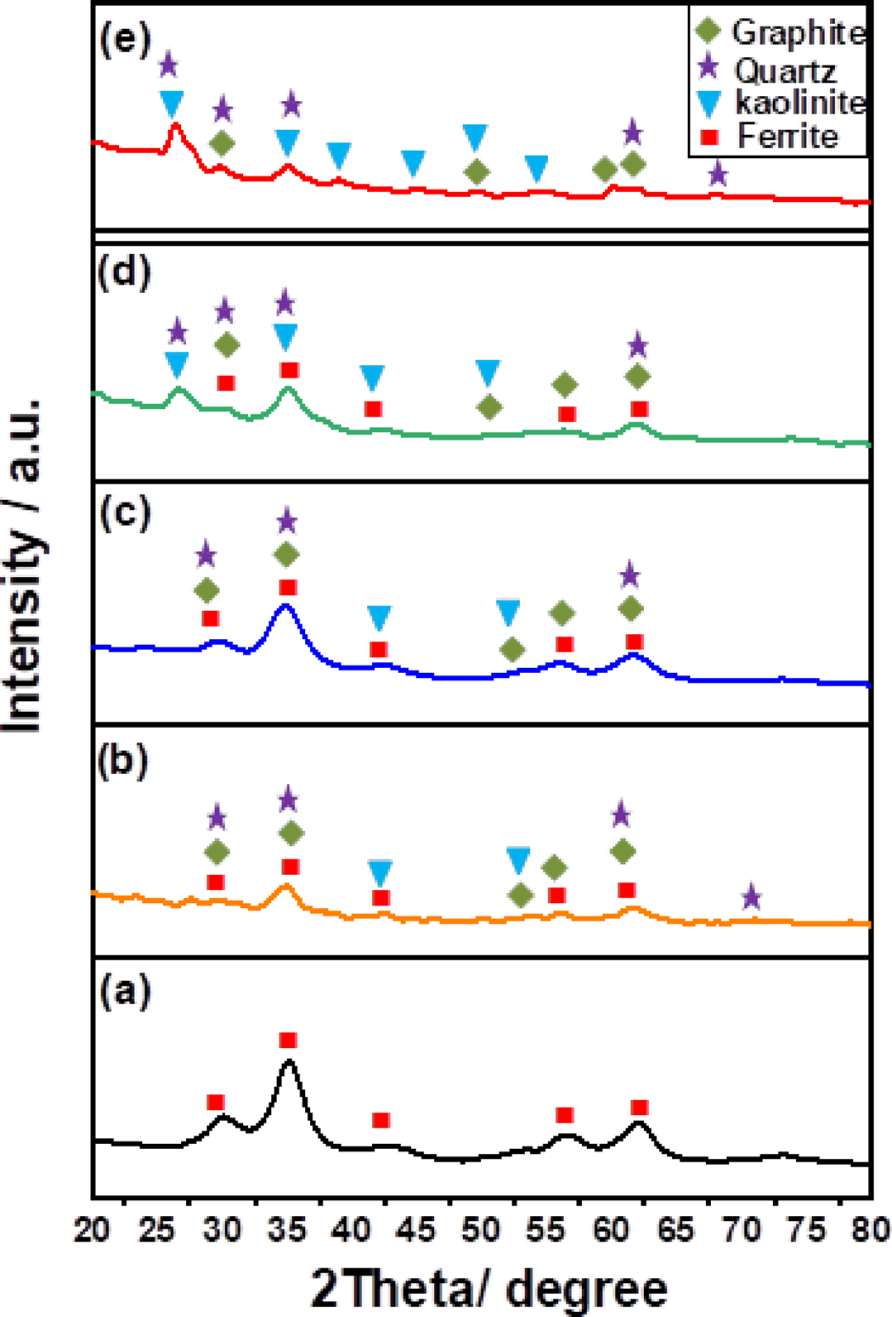
XRD of the (**a**) F-Zn (**b**) F-Zn/AS400 (1:1) (**c**) F-Zn/AS400 (2:1) (**d**) F-Zn/AS400 (1:2) (**e**) AS400.

Figure 2 shows the results of an XRD diffraction investigation of F-Zn, and its composites with AS400 (F-Zn/AS400 (1:1), F-Zn/AS400 (2:1) and F-Zn/AS400 (1:2)). The F-Zn crystal planes (220), (311), (400), (511) and (440), are indexed to the characteristic diffraction peaks at 2θ of 29.9°, 35.2°, 42.8°, 56.5°and 62.1°, (JCPDS 002-4496)^22^. The AS400 also exhibited a good crystalline phase, as evidenced by the well-defined sharp diffraction peaks in its XRD pattern. These peaks were identified as the contributions of complex phases, such as graphite (C), quartz (SiO_2_) (JCPDS 46-1045), which was the predominant component of the sludge and kaolinite (Al_2_Si_2_O_5_(OH)_4_, (JCPDS 29-1488)^23,24^.

Quartz (silicon oxide, SiO_2_), which has a hexagonal crystal shape, is the primary crystalline inorganic substance in this type of sludge^25^.

The suspended materials like sand and clay in the raw water could be the source of such quartz particles because aluminum sulfate is the only coagulant used in this water treatment facility. All the prepared composites also contain graphite (C), which has a hexagonal crystal structure^25^.

Therefore, the presence of distinctive AS400 and F-Zn peaks on F-Zn/AS400 indicated that all the three composites were successfully synthesized. The average crystallite size (D) was determined using the Debye–Scherrer equation as shown below.

3 $${\text{D}} = \frac{{0.9\, {{\varvec{\uplambda}}}}}{{\upbeta \,\cos \,\uptheta }}$$

D is the expected crystalline size, θ is the diffraction angle, β is the full width at half maximum (FWHM) of catalysts, and λ is the wavelength of the X-ray source (0.154 nm) utilized in XRD^26^. The calculated average crystal sizes for F-Zn, F-Zn/AS400 (1:1), F-Zn/AS400 (2:1) and F-Zn/AS400 (1:2) were 4.456 nm, 6.512 nm, 4.830 nm, and 6.684 nm, in that order. The introduction of F-Zn particles into composites may have contributed to the lack of significant crystal size enhancement observed in the data^22^.

#### FTIR spectra

**Fig. 3:**
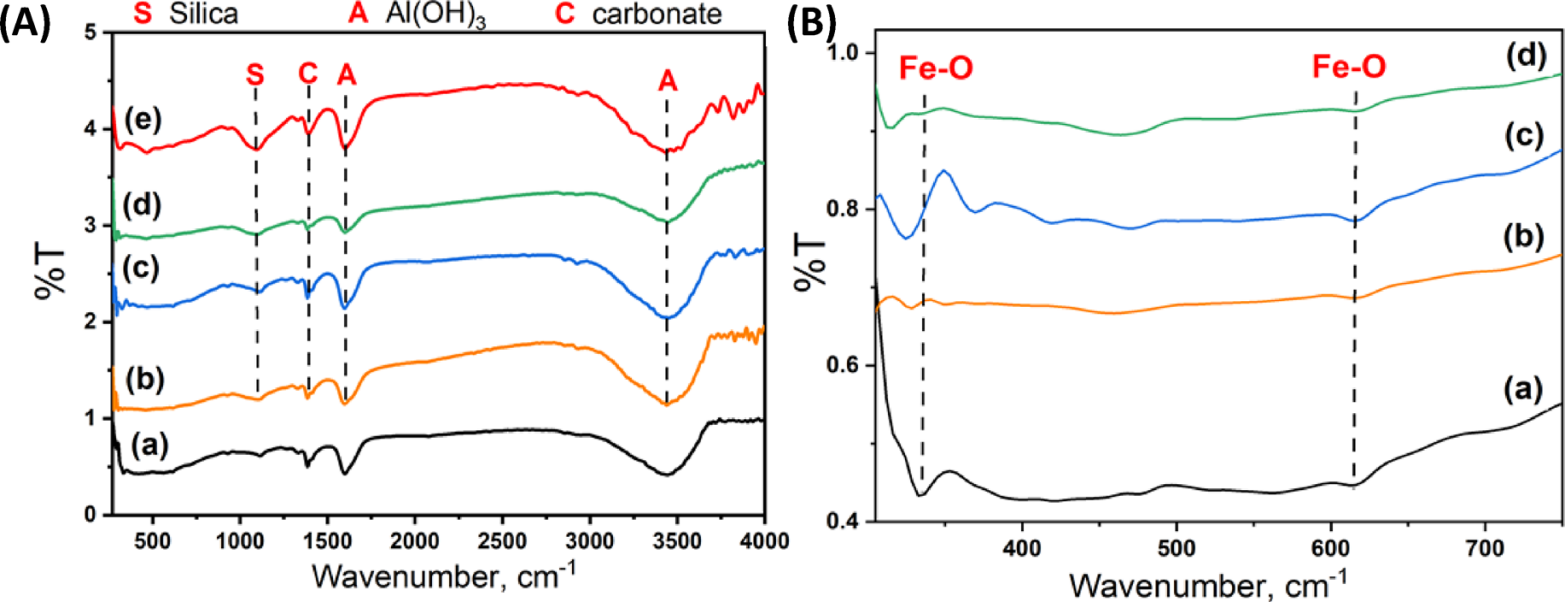
FTIR of the (**a**) F-Zn (**b**) F-Zn/AS400 (1:1) (**c**) F-Zn/AS400 (2:1) (**d**) F-Zn/AS400 (1:2) (**e**) AS400.

FTIR spectroscopy is also used to characterize the surface functional groups of the prepared samples, AS400, F-Zn, and their combinations as composites with AS400 and the results are displayed in Fig. 3A,B. The data exhibited in Fig. 3Aa–e demonstrates that two unique peaks originating from oxygen bonding are displayed in F-Zn structures. The presence of strong intensive peaks are frequently observed by absorption bands at about 613 cm^−1^, which is due to the stretching vibration of the tetrahedral structure of the metal–oxygen interaction. Furthermore, the peaks at the range of 419 to 464 cm^−1^, weak intense peaks were identified that are related to the metal–oxygen bond in the octahedral structure. The existence of free hydroxyl on the carbon surface is suggested by the broad band at 3430 cm^−1^ for each spectrum that corresponds to the O–H stretching vibration mode of hydroxyl, or surface-adsorbed water^27^.

The peak observed at 1088 could be related to H–o–H water molecules’ vibrational bending at the F-Zn and their composites with AS400^27^ or the Si–HO–HSi stretching vibrations (silanol), indicating the presence of quartz in AS400 and the three composite samples. The Si–HO–HSi stretching band’s position and intensity provide information about the type of silicate network^28^. The Si–HO–HAl and Al–HO–Al deformation is appeared at 3820 cm^−1^ implied the characteristic bands of kaolinite^21^. However, at the wavenumber of about 1590 cm^−1^ the absorption band of carbonate as appeared in the graph. The Al–O stretching vibration is signifying the bending vibration of water molecules chemically bound to Al(OH)_3_, and the OH stretching of Al(OH_)3_ are all correlated with the absorption bands of Al(OH)_3_ at 500, 1602, and 3438 cm^−1^, respectively^28^.

#### SEM

**Fig. 4:**
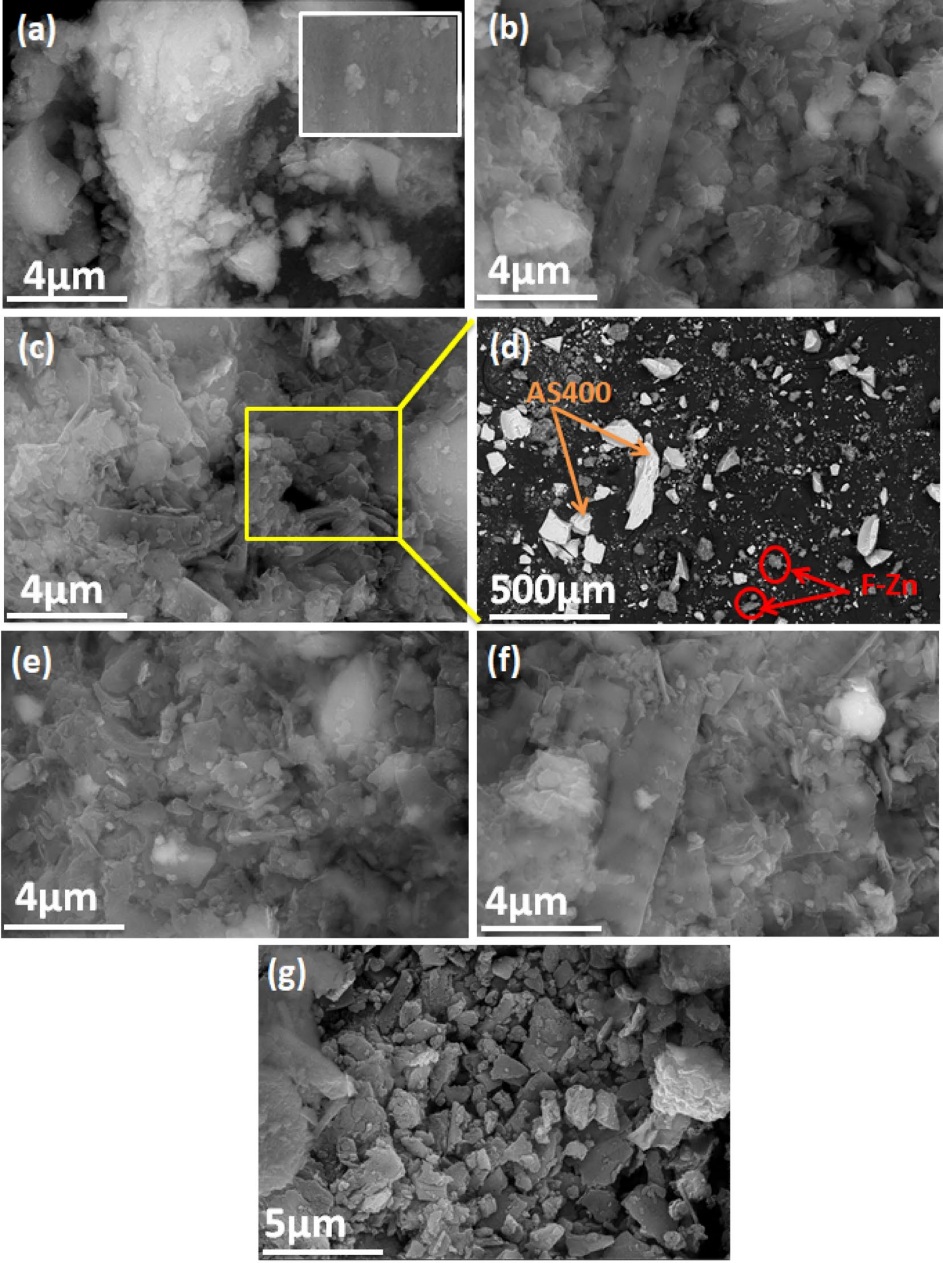
SEM image of (**a**) F-Zn (**b**) F-Zn/AS400 (1:1) (**c**) F-Zn/AS400 (1:2) scale 4 μm (**d**) F-Zn/AS400 scale 500 μm (1:2) (**e**) F-Zn/AS400 (2:1) (**f**) F-Zn/AS400 (2:1) after adsorbed dye and (**g**) AS400.

For the object of exploring the morphology and surface of the prepared nanoparticle of F-Zn and their composite materials, SEM morphology is applied and the images are displayed in Fig, 4. According to the images, F-Zn (Fig. 4a), the micrograph showed a significant quantity of semi-spherical and rough particles that formed severe aggregation because of the small particles’ nanoscale surface effect and their magnetic nature^23^. Figure 3g shows the porous structure of the prepared AS-400 which composed of semi-hexagonal sheet and maintains silica’s amorphous nature. The SEM micrograph of the alum F-Zn AS400 composite exhibited a semi-hexagonal sheet of alum sludge with an augmented semi-spherical quantity of ferrite nanocrystals (Fig. 4b–e)^29^. The roughness and uneven surfaces provide better adsorption sites for pollutants as compared to smooth and even surfaces. The increasing surface roughness provides improved fractional dimensions which result in enhanced surface interaction as compared to planer surfaces^22^. SEM pictures obtained after adsorption (Fig. 4f) provided a visual depiction of the sludge containing the dye molecules. After adsorption, the material’s rough surface was visibly smoothed out to become more extended, and its morphology took on the appearance of a petal. The expansion of the AS-Sorbent surface, which is rebuilding its structure, may be seen as a result of the dye molecules adsorbed on the AS-Sorbent solid material entering the surface to the substance’s pores^25^.

#### TEM

**Fig. 5:**
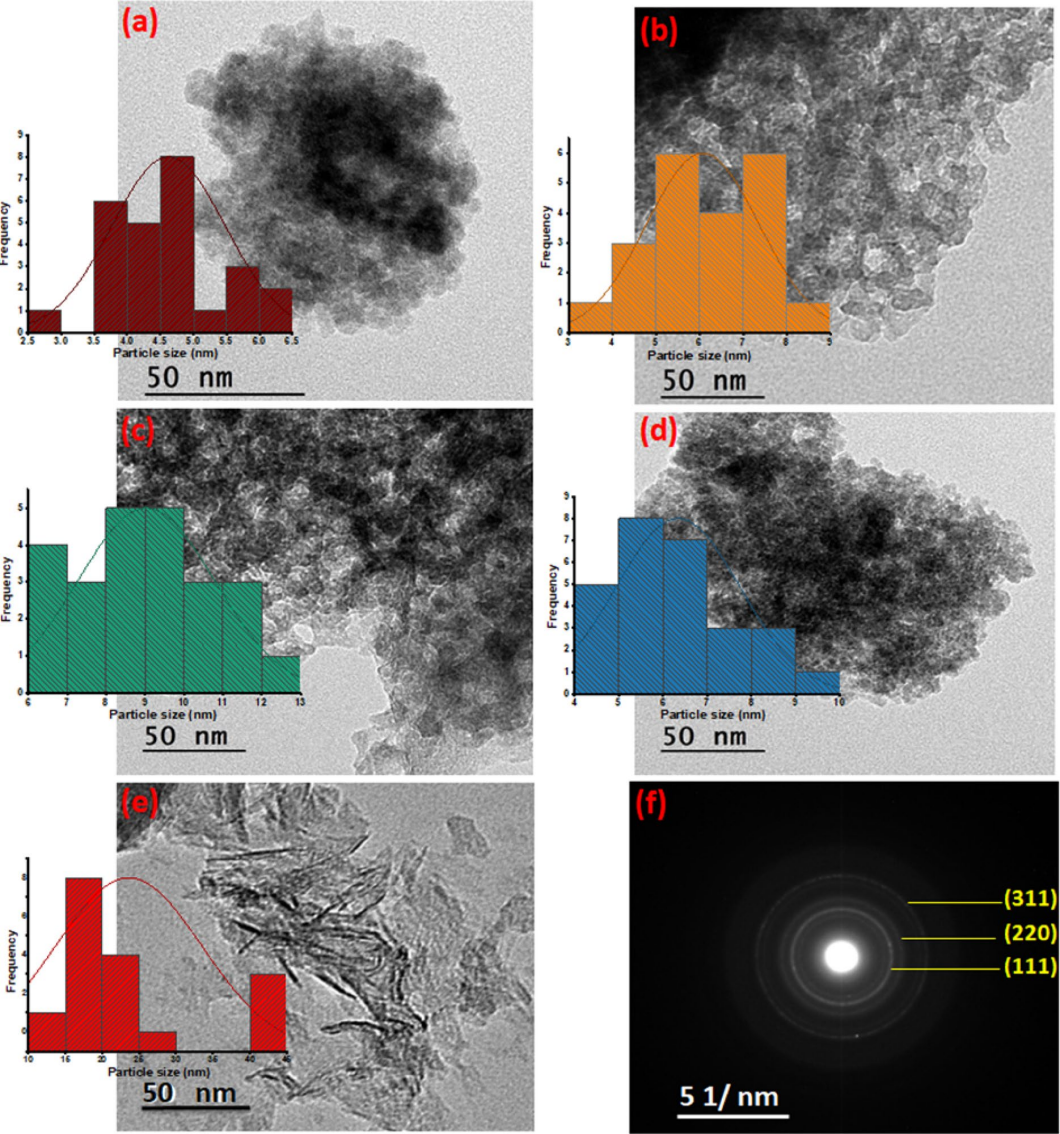
TEM image of (**a**) F-Zn (**b**) F-Zn/AS400 (1:1) (**c**) F-Zn/AS400 (1:2) (**d**) F-Zn/AS400 (2:1), (**e**) AS400 and (**f**) SAED pattern of F-Zn nanoparticle.

Figure 5 exhibited the morphology and crystallite size of F-Zn, F-Zn/AS400 (1:1), F-Zn/AS400 (1:2) and F-Zn/AS400 (2:1) composites prior and after adsorption test through TEM images. It is observed from (Fig. 5a), the prepared ferrite is displaying spherical or semi-spherical nanoparticles and the particles are agglomerated together. Such findings might be explained by the interaction of magnetic nanoparticles together due to the high surface energy^30^. However, for the composite materials containing different amounts of ferrite nanoparticles and calcined alum sludge that are displayed in Fig. 5b–d, spherical agglomerated particles are appeared that is signifying the presence of F-Zn. Furthermore, the spherical particles embedded in the material are increased with the ferrite nanoparticles increase as seen in Fig. 5d. Furthermore, the ferrite was evenly distributed across the composite surfaces, which is also confirmed by the above-mentioned SEM pictures. It is evident that the AS400 is a mixture of hexagonal-like particles and phases rod-like (Fig. 4e)^28^.

Additionally, using Image-J software version 154 assessed the particle size distribution histograms for the as-synthesized adsorbents. The results displayed in the inset of Fig. 5 showed each particle size distribution of the solo materials and the augmented composite substances in various proportions. As exhibited in (Fig. 5a–e), the average crystallite sizes of the F-Zn, F-Zn/AS400 (1:1), F-Zn/AS400 (2:1) and F-Zn/AS400 (1:2) are signified as 4.962 nm, 6.500 nm, 6.338 nm and 9.031 nm, respectively. Such results verify the attained data from the XRD patterns. Further, Fig. 5f displays the samples’ selective area electron diffraction (SAED) patterns. It is discovered that a pure F-Zn with great crystallinity is polycrystalline. Additionally, a Debye ring pattern is obtained for the bright area, indicating the polycrystalline structure of the materials^31^.

#### VSM and BET studies

**Fig. 6:**
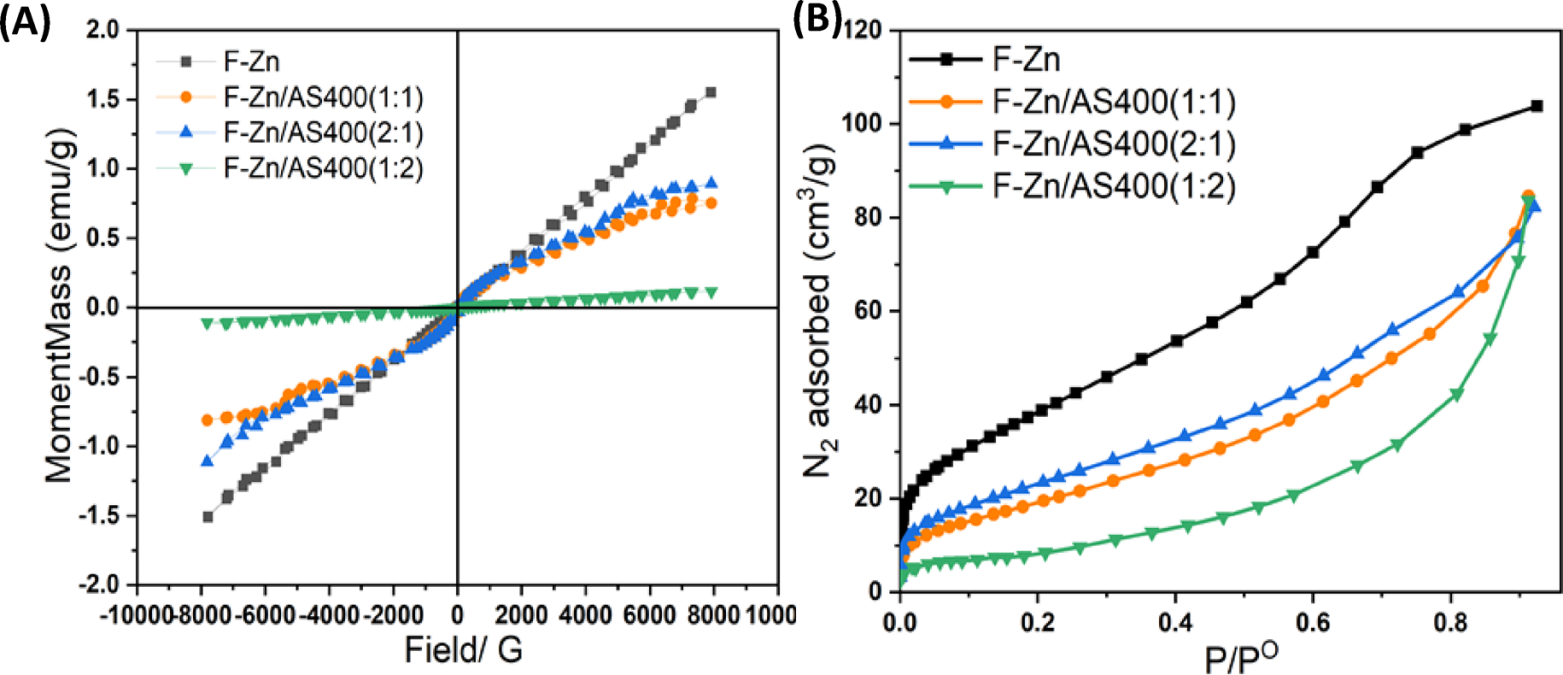
(**a**) Hysteresis loops, and (**b**) Nitrogen adsorption–desorption isotherms of F-Zn, F-Zn/AS400 (1:1), F-Zn/AS400 (2:1), and F-Zn/AS400 (1:2).

The magnetism of the proposed adsorbent materials is determined via the use of vibrating sample magnetization (VSM) technique and the results are presented in Fig. 6A. The saturation magnetizations of F-Zn, F-Zn/AS400 (2:1), F-Zn/AS400 (1:1) and F-Zn/AS400 (1:2) are exhibited 1.539 emu/g, 0.8953 emu/g, 0.7504 emu/g and 0.1154 emu/g, respectively. the data displays that the percent of AS400 and its increase in the composite augmented with F-Zn, which results in lesser magnetization values in comparison to solo F-Zn. This could be attributed by the non-magnetic nature of AS400. But, it is noteworthy to mention that such composites still possess magnetic characteristics. Consequently, the VSM investigations verified that AS400 is embedded with F-Zn nanoparticles and the material still have magnetic properties even though it is lower than that of the pure ferrite material.

To add up, The BET surface area is calculated and displayed in Fig. 6B and Table 1. The physisorption of non-reactive gases (such N_2_) provides the data needed to calculate surface area, pore volume, and also pore interior design. As shown in the inset of Fig. 6B, the produced composites all exhibit a strong absorption of N_2_ at low relative pressure, indicating that type II isotherms through the IUPAC classification were present in all samples, which is related to substances that are microporous and have pores 4.29, 6.53, 12.09 and 5.57 nm for the F-Zn, F-Zn/AS400 (1:1), F-Zn/AS400 (1:2) and F-Zn/AS400 (2:1) respectively^27^. Both monolayer and multilayer adsorption on the surface were displayed by the graphs in the adsorbents’ isotherms^32^.

**Table 1:** Values of BET measurements pore volume (Vp) and pore diameter (Dp) of the F-Zn ferrite and their composites with AS400 nanomagnetite materials.

Material	Dp/(nm)	Vp/(cm^3^/g)	S_BET_/(m^2^/ g)
F-Zn	4.29	0.16	126.3
F-Zn/AS400(1:1)	6.53	0.13	76.2
F-Zn/AS400(2:1)	12.09	0.13	82.0
F-Zn/AS400(1:2)	5.57	0.12	52.6

Typically, H3 hysteresis loops imply aggregated particles, and they were present in all the materials. A steep adsorption branch is present at a relative pressure of unity is due to significant interparticle porosity.

The volume adsorbed by F-Zn is much more than the AS400 and their composites indicate higher interparticle porosity F-Zn. The higher interparticle porosity of F-Zn leads to higher total pore volume (0.1606 cm^3^/g). F-Zn with a larger surface area (126.26 m^2^/g) exhibits increased nitrogen adsorption^33^. The mineralogical compositions of the F-Zn impact their specific surface area and nitrogen adsorption properties^24^. Table 1 provided a summary of the surface area values derived from the BET analysis. Such findings are in accordance with the overall pattern that as the crystallite size decreases as the surface area increase. The surface area played an important role in the adsorption process. More the surface area of ferrite NPs and their composites, more the active sites available for adsorbate attachment and hence higher was the adsorption.

#### EDX analysis

**Fig. 7:**
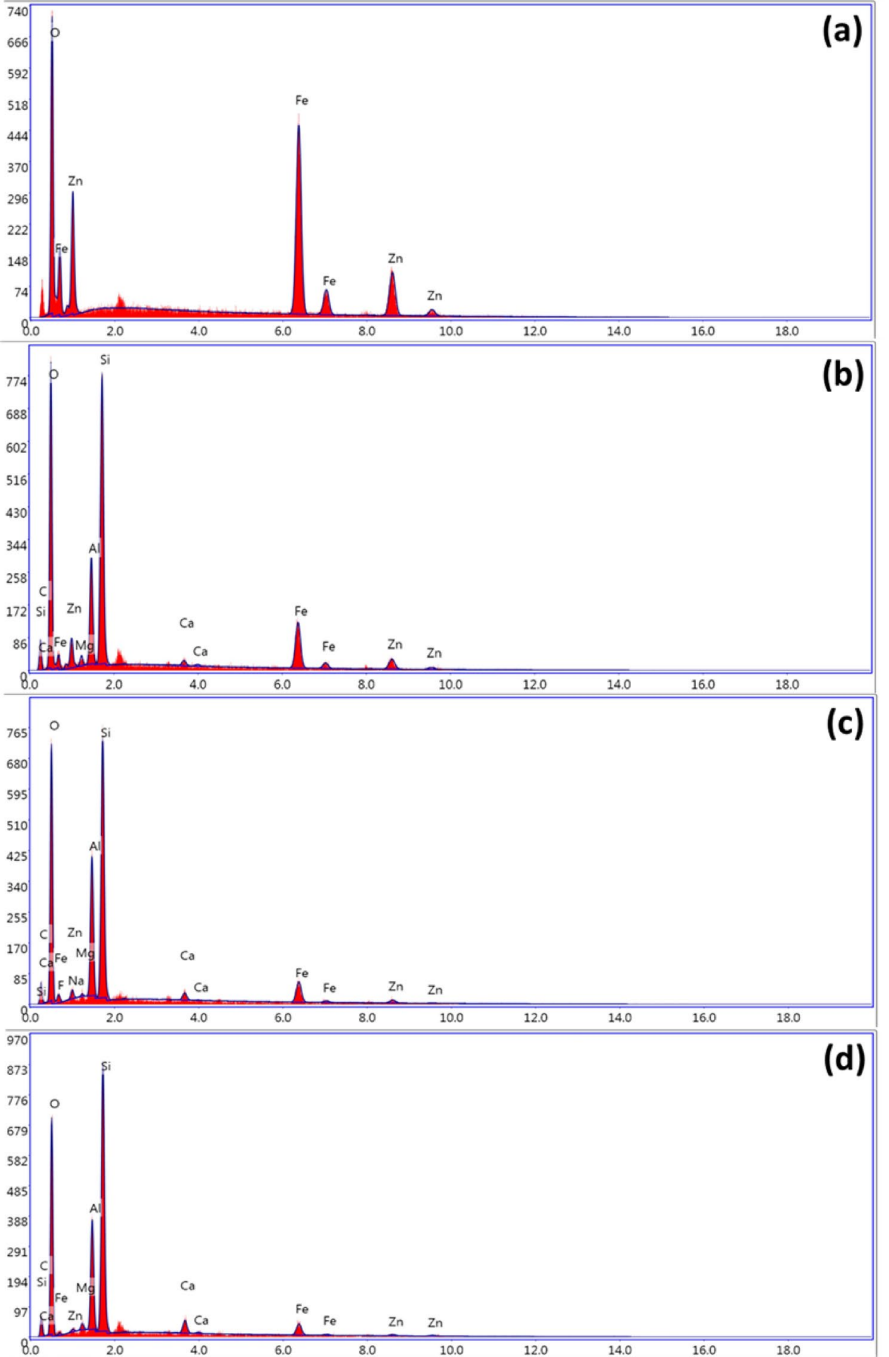
EDX analysis of (**a**) F-Zn (**b**) F-Zn/AS400 (1:1) (**c**) F-Zn/AS400 (2:1) (**d**) F-Zn/AS400 (1:2).

Energy-dispersive X-ray analysis (EDX) is investigated to determine the elemental composition in F-Zn and their composites augmented with AS400 as exhibited in Fig. 7. Pristine F-Zn possesses zinc (Zn), iron (Fe) and oxygen (O) that are indicating the formation of F-Zn (Fig. 7a). Also, there are another peaks observed after modification with the addition of AS400 such as silicon (Si), Aluminum (Al), carbon (C) and small traces of magnesium, sodium and calcium as displayed in Fig. 7b–d. The highest content was discovered to be oxygen because there were a lot of oxide minerals present. Silicate and aluminosilicate minerals are the source of silicon and aluminum. The carbon material in the water sludge was derived from the soil organic matter (SOM), which was created by microbial activity during sludge storage^24^.

### Adsorption test of indigo carmine dye

#### Effect of isotherm time

**Fig. 8:**
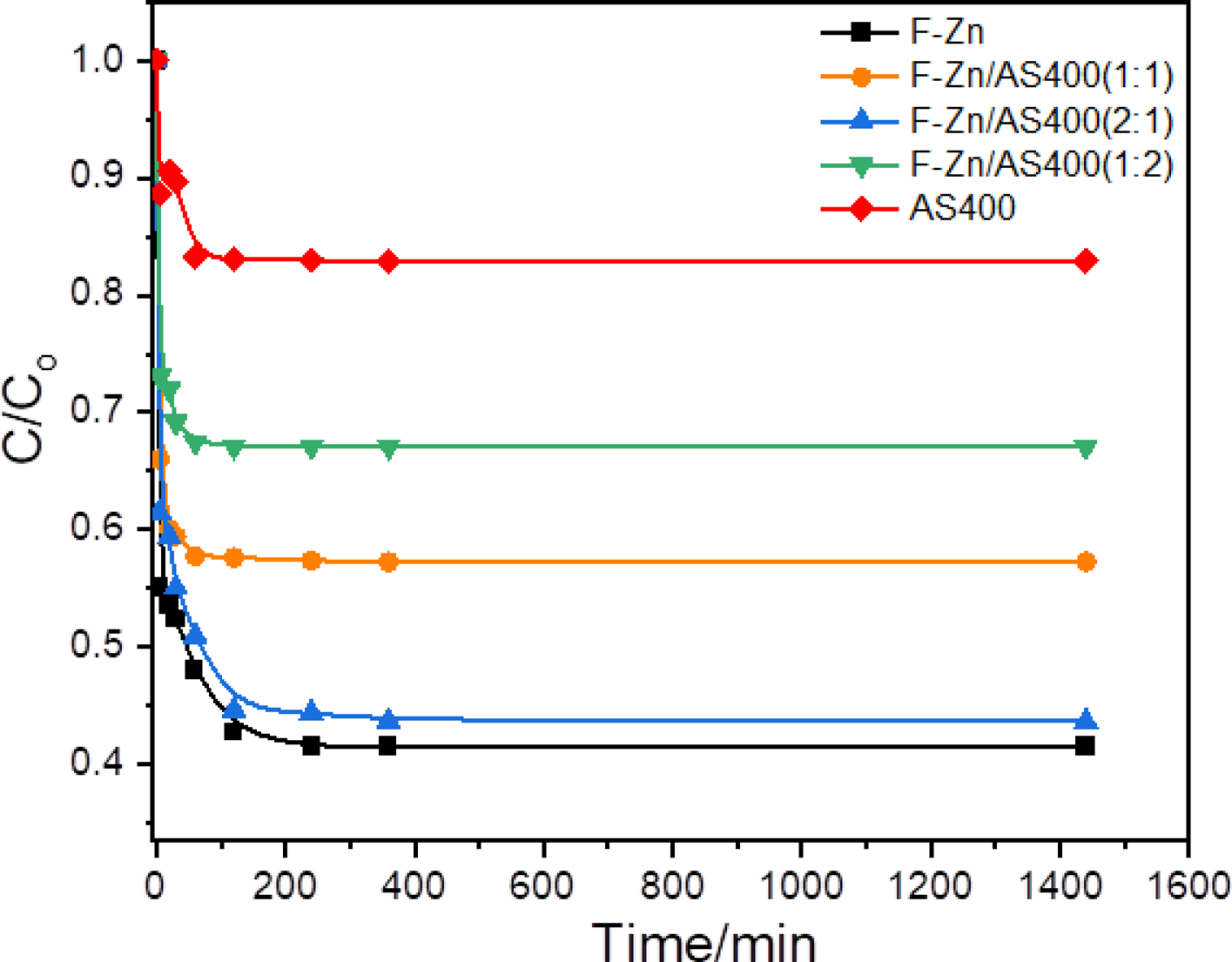
Effect of adsorption contact time on the IC dye uptake with different adsorbents (IC 20 ppm; adsorbent dose 0.5 g/L; pH 6 and 298 K).

Preliminary, the adsorption equilibrium time is essential to be examined prior creating the adsorption matrix. In this regard, the time-profile of IC dye adsorption is investigated by the use of various adsorbents, F-Zn, F-Zn/AS400 (1:1), F-Zn/AS400 (2:1), F-Zn/AS400 (1:2) and AS400 at room temperature. According to the data displayed in Fig. 8, F-Zn exhibited the highest dye adsorption capacity overall, with the majority of the dye adsorbed within the first initial 2 h of contact time. Surprisingly, no excess IC dye is absorbed for the all applied adsorbents during an extended contact adsorption time longer than the first 2 h of contact time. Thus, noticeably, for all the applied adsorbents, there is no further IC uptake with the prolonged reaction time than 2 h. This could be attributed by the accessible active sites of such adsorbent materials are become saturated with IC molecules. Hence, further contact time not leading to the adsorption of extra dye molecules.

Overall, F-Zn in comparison to the pristine F-Zn or AS400 augmented with F-Zn in varied combinations percent displayed the highest adsorption capacity. Also, it is essential to mention that the presence of AS400 with F-Zn declines the adsorption capacity. The adsorption capacity is declined with the amount of AS400 increase in the composite the results displayed that the adsorption capacity is ranged from 19.86, 15.76 and 12.17 mg/g for F-Zn/AS400 (1:1), F-Zn/AS400 (1:2) and F-Zn/AS400 (2:1), respectively. However, the pristine F-Zn is displayed 20.75 mg/g and the solo AS400 adsorbent exhibited 15.72 mg/g of adsorption capacity. Such results verify the role of the combination effect of F-Zn and AS400 as a composite in enhancing the IC dye removal efficiency. It was also shown that the adsorption capacity increase with increasing the percent of F-Zn in three composites with AS400. Such results are in accordance with the data displayed from the BET analysis that verified the prepared ferrite has the largest BET surface area (126.26 m^2^/g) as well as their microporous nature of the adsorbent. It is also appeared that the high adsorption of F-Zn was facilitated by the unrestricted access of active sites, or oxygen anions, at the formed surface of ferrite nanoparticles^8^. Also, higher surface area permits excess available adsorption sites to be used and hence increasing the adsorption tendency and the adsorption capacity thereby increases. Furthermore, it is noteworthy to mention that the existence of graphite in all AS400 campsites also supports the adsorption process since it is highly effective in reactive dye uptake. Ultimately, the equilibrium time is estimated to be 2 h and fixed in the further experiments.

It is noteworthy to mention that the presence of ferrite in the composite contains AS400 increases the adsorption capacity and possess the merits of making the adsorbent is recyclable. Such results are in accordance with the previous work reported in literature^34,35^ in treating dye solutions using ferrite nanoparticles and aluminum-based waste.

#### Effect of adsorption variables on the adsorption uptake

##### Effect of adsorbent dose

**Fig. 9:**
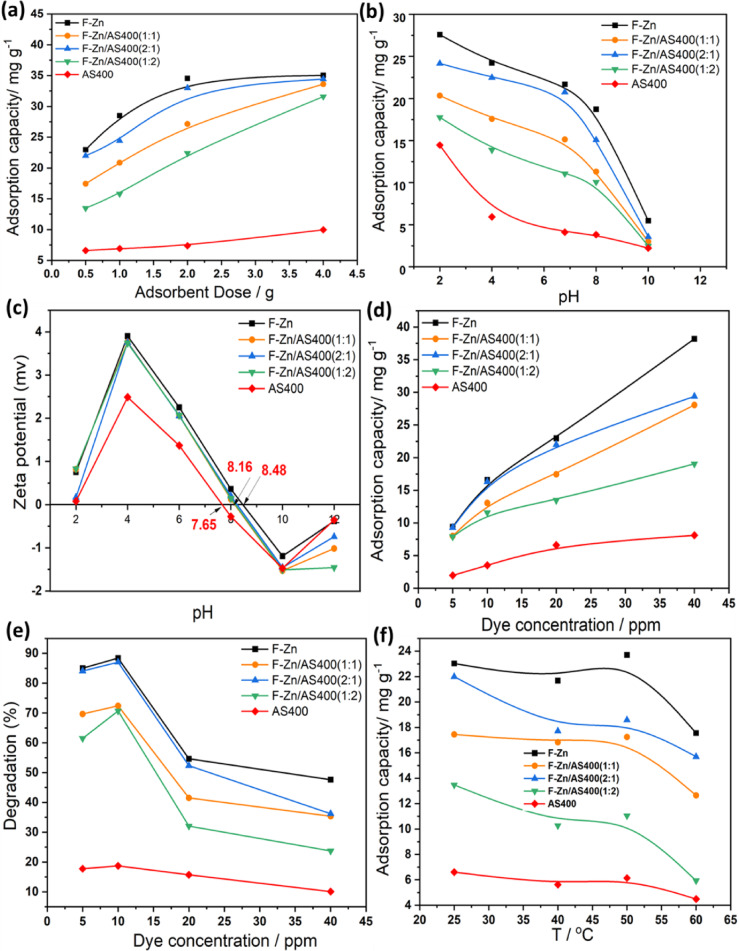
Effect of operating parameters on adsorption capacity (**a**) effect of adsorbent dose (IC 20 ppm; pH 6 and 298 K), (**b**) effect of pH (IC 20 ppm; adsorbent dose 0.5 g/L and 298 K), (**c**) zeta potential of adsorbents and (**d**), (**e**) effect of IC concentration (adsorbent dose 0.5 g/L; pH 6 and 298 K) and (**f**) effect of temperature (IC 20 ppm; adsorbent dose 0.5 g/L and pH 6).

(IC) dye uptake for the adsorbents ranged from the solo systems F-Zn and AS500 or the composite systems F-Zn/AS400 in a proportions of (1:1), (1:2) and (2:1) dosages used, whereas other operating variables remained fixed, are displayed in Fig. 9a. In this regard, different amounts of F-Zn and their composite with AS400 from 0.5 to 4.0 g/L were added to the dye solution. It could be seen that the adsorption capacity of Indigo carmine enhanced with an increase in sorbent doses for all the studied system. Hence, the dosage of F-Zn and their composite with AS400 from 0.5 to 4.0 g/L, IC dye adsorption capacity enhanced. More available sites could attribute this for adsorption process is accessible^7^. This results is in accordance with the previous reports of Mangood et al., they verified that the adsorption capacity increases when adsorbent dosage increase from 0.5 to 5.0 g/L^21^

##### Effect of pH

For the object of maximizing the adsorption uptake, the influence of the pH on the adsorption capacity should be also tested. pH is categorized as one of the most crucial factors influencing the adsorbent surfaces that affects its characteristics and also affects the presence of appropriate functional groups on the adsorbate molecules. Generally, the molecules of the adsorbent and adsorbate possess a variety of surface functional groups that might be protonated or deprotonated according to the aqueous medium pH value. Such phenomena could be helps the contaminant molecules to modulate the electrostatic contact between the opposite charges, which thereby improves the effectiveness of pollutant removal^36^. Hence, experiments are conducted at various initial pH values ranged from acidic (2.0) to alkaline range (10.0) by fixing other parameters and the data displayed in Fig. 9b.

It can be noticed from the experimental results displayed in Fig. 9c that the higher adsorption capacity was achieved at pH value corresponding to 2.0. But, increasing the pH value from (2.0 to 10.0), the adsorption capacity is declines. This could be illustrated by the a zero-charge surface (pHpzc) of F-Zn, AS400 and their composites is determined at a pH value from 7.65 to 8.48 (as shown from Fig. 9c. thereby, this could be signifies that the pH value more than such values, the prepared adsorbent surfaces are negatively charged and in contrary the surface is positively charged at pH value less than these values. Additionally, Indigo carmine is categorized as an anionic dye, hence there is attraction forces appear between the dye and positively charged surface in the acidic medium. However, the adsorption capacity is declined at pH values higher than 9.5. This is can be illustrated by the available negatively charged hydroxyl groups (^-^OH) in the reaction medium and also the presence of the anionic dye. In this regard the negatively charged hydroxyl groups (^-^OH) competing the dye molecules to be adsorbed in the surface of the adsorbent material due to surface interaction^36^. The result is overall decline in the adsorption capacity for the all applied samples from 27.57 to 5.47, 20.34 to 2.99, 24.16 to 3.65, 17.75 to 2.47 and 14.45 to 2.21 mg/g, for F- Zn, F-Zn/AS400 (1:1), F-Zn/AS400 (2:1) and F-Zn/AS400 (1:2), and AS400, respectively, from acidic to alkaline medium, respectively. Such result of superior capacity of adsorption that is corresponding to the acidic pH is previously verified by other researcher in treating different pollutants^37^. However, other researchers exposed in their investigation, an alkaline pH are favored.^38^. Hence, the solution’s pH value affecting in diminishing or may be increasing the attraction forces between the adsorbent material and the adsorbate and hence the overall adsorption uptake is affected.

##### Effect of indigo carmine dye concentration

For the object of real life applications, it is essential to study the pollutant loads. One of the main factors affecting aquatic life is the concentration of dye in the effluent water. Hence, the dye load on the treatment efficiency and adsorption uptake is investigated in the range of 5 to 40 ppm. The experimental findings indicate that adsorption capacity increases with the increase in initial dye concentration from 5 to 10 ppm and recorded as an increase from 8.49 to14.96, 7.23 to 11.79, 8.39 to14.73, 7.13 to 10.44 and 1.77 to 3.17 mg/g, for F-Zn, F-Zn/AS400 (1:1), F-Zn/AS400 (2:1) and F-Zn/AS400 (1:2), and AS400 adsorbents, respectively, as displayed in Fig. 9d. This might be associated to the increase in the driving forces between the dye molecules and the adsorbent material with the increase in the initial IC dye concentration. Also, the increase in the available adsorbate molecules could help in interacting such molecules with available sites on the surface of the prepared adsorbents. Thereby, the result is a steady increase in the adsorption capacity of the F-Zn and their composites with AS400 is signified^39^. However, a decline in the dye removal efficiency is highlighted by further increase in the IC dye concentration after the increase higher than 10 ppm. As exhibited in (Fig. 9e, increase in the dye concentration excess than 10 ppm to 40 ppm and the corresponding adsorption capacity recorded as 34.51, 25.64, 26.25, 17.21 and 7.32 mg/g for F-Zn, F-Zn/AS400 (1:1), F-Zn/AS400 (2:1) and F-Zn/AS400 (1:2), and AS400 adsorbents, respectively when the dye concentration is 40 ppm. Such result could be linked to when the IC dye concentrations is low, the dye molecules in the solution interacts with the adsorbent’s available sites. Also, according to the above-mentioned data, the increase in the dye concentration motivating in the increase in the driving forces between dye and adsorbent and thereby increases the dye uptake. But, at a certain limit, the available active sites on the adsorbent material become saturated with the dye molecules. Hence, the too high concentration of the adsorbate is not favorable because the vacant sites reach their saturation point and limiting the dye uptake. Such result is in good agreement with previous work cited in literature^40^.

##### Effect of temperature

According to this literature^41^, the temperature change on the adsorption system has a high influence and impact. Such effectiveness could be signified as one of the following: (i) changing the solubility and viscosity of dyes in solution; (ii) expanding the adsorbent’s internal structure; or (iii) harming the adsorbent’s active sites^41^. Thus, temperature effect on IC dye adsorption capacity is assessed. According the experimental work displayed in Fig. 9 (f), the temperature by its increase from room temperature and checked from 40 to 60 °C. The data exhibited that adsorption capacity of the prepared samples is changed from (23.03 to 21.68), (17.44 to 16.83), (21.99 to17.70), (13.47 to 10.27) and (6.61 to 5.62) mg/g, for the adsorbents of F-Zn, F-Zn/AS400 (1:1), F-Zn/AS400 (2:1) and FZn/AS400 (1:2), and AS400, respectively for the temperature increase from room temperature to 40 °C. The temperature increase results in the adsorbate molecules diffuse in more efficient way over the exterior boundary layer and interim adsorbent molecule holes^42^. But, further increase in the temperature up to 50 °C results in further decline on the adsorption capacity for all the prepared adsorbents. This could be illustrated by the diffusion rate of ions increased and the adsorption capacity drops above this temperature. These result of increasing the adsorption uptake with a temperature increase at a certain temperature limit, but, beyond such temperature (313 K) is thereby declined is in agreement with the previous work stated in the literature^43^.

##### Recyclability test

To verify the novel material based on waste alum sludge sustainability, it is essential to check its recover and reuse facility. In this regard, F-Zn/AS400 (1:1) material that l is easily recovered via the presence of ferrite due to its good magnetic properties is collected after fresh use and then subjected for successive washing with distilled water. Then, the collected washed sample is then exposed for oven drying at 105 °C. the sample is subsequently used for wastewater treatment and the removal of IC dye efficiency in the first use and after regeneration is compared. The experimental results displayed a reduction in the removal rate from 54 to 35%. This reduction in the removal efficiency is associated of vacant active sites on the adsorbent surface with the occupation of dye molecules. Even through the material is washed for regeneration it might be still need further activation that require further study. But, it is noteworthy to mention that although a simple regeneration is achieved during the current work the adsorptions/desorption cycle is still show a reasonable treatment compared to the first use of the material.

#### Kinetic study

Three steps are involved in the elimination of undesirable pollutants from the synthesized magnetic adsorbent surface: the transfer of dissolved pollutant molecules from the liquid phase to the solid adsorbent’s surface, the actual adsorption on the solid surface, and the intraparticle diffusion of adsorbate molecules into the adsorbent’s pores. When contrasted with the internal intraparticle diffusion, the outward diffusion of dye molecules on the adsorbent surface step typically happens exceedingly quickly. The adsorption equilibrium usually takes place in a few minutes. The internal diffusion mechanism is predominant and regulates the reaction rate when adsorption equilibrium takes a long time to reach. Since it may be used to estimate the rate of pollutant removal and comprehend the adsorption mechanism. There are numerous models that can be used to explain the mechanism of pollution removal, such as pseudo-first-order, and pseudo-second-order models^33^.

According to pseudo-first-order kinetic model (Eq. 9), each dye molecule is adsorbed at a single sorptive site in the adsorbent material (Eq. 4).

4 $$\log (q_{e} - q_{t} ) = \frac{{k_{1} }}{2.303}{\text{t}} + \log \,q_{e}$$

where k_1_ is the adsorption pseudo-first-order rate constant, q_e_ is the quantity of dye adsorbed at equilibrium (mg/g), and q_t_ is the amount of dye adsorbed at time t (mg/g).

The pseudo-second-order kinetic model (Eq. 10) assumes that the square number of unoccupied sites and the occupation rate of sorption sites are proportionate.

5 $$\frac{t}{{q_{t} }} = \frac{1}{{K_{{2q_{e}^{2} }} }} + \frac{1}{{q_{e} }}{\text{t}}$$

where k_2_ is the adsorption pseudo-second-order rate constant.

**Fig. 10:**
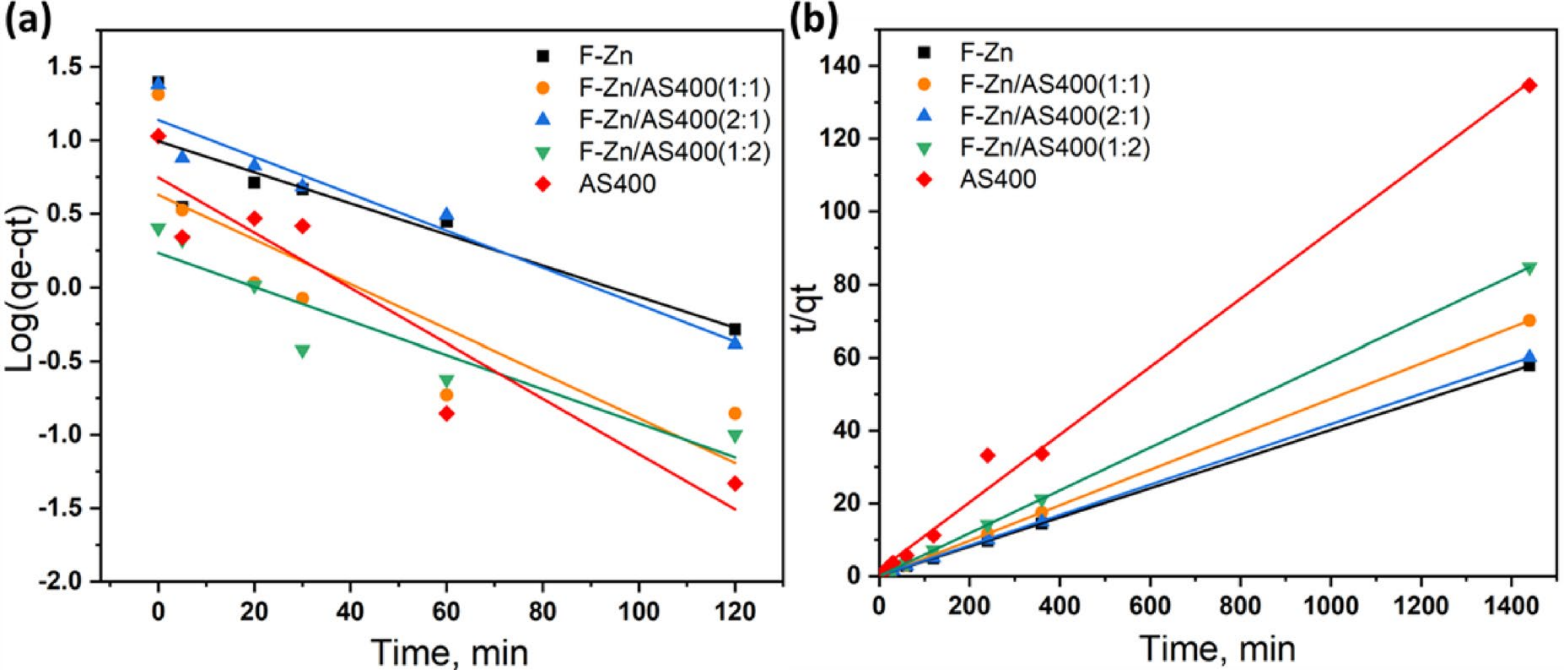
Kinetic modeling plot of IC adsorption (**a**) first order kinetics and (**b**) second order kinetics.

The experimental data of IC removal on the nanoadsorbent are applied to the kinetic models and plotted (Fig. 10A,B) according to the first order kinetics and second order kinetics, respectively to select the best model fit. It is estimated from the correlation coefficient (R^2^) that the inadequate model fit is the pseudo first order equation since the correlation coefficient is low. Such values are tabulated in Table 2, where there was a significant difference between the calculated and experimental q_e_ values. This estimates that the pseudo-first order is not fitting the experimental data well. But, the correlation coefficient data that signifies the pseudo-second-order kinetics is high (0.99–1.0) that means the IC dye adsorption on adsorbent nanoparticles can be described by pseudo-second-order kinetics and well fir the experimental data.

**Table 2:** Parameters corresponding to the models for adsorption kinetics.

Model parameters	F-Zn	F-Zn/AS400(1:1)	F-Zn/AS400(2:1)	F-Zn/AS400(1:2)	AS400
Pseudo-1st order					
${\text{q}}_{\text{e}}$(mg/ g)	9.87	4.26	13.75	1.71	5.59
k_1_(min^−1^) × 10^–2^	2.44	3.49	2.89	2.66	4.33
R^2^	0.77	0.71	0.93	0.87	0.88
Pseudo-2nd order					
${\text{q}}_{\text{e}}$(mg/ g)	24.97	20.55	24.06	17.01	0.721
K_2_(min^−1^) × 10^–2^	1.04	5.63	0.79	3.59	10.75
R^2^	0.99	1.0	0.99	1.0	0.99

#### Adsorption isotherm

##### Langmuir isotherm

**Fig. 11:**
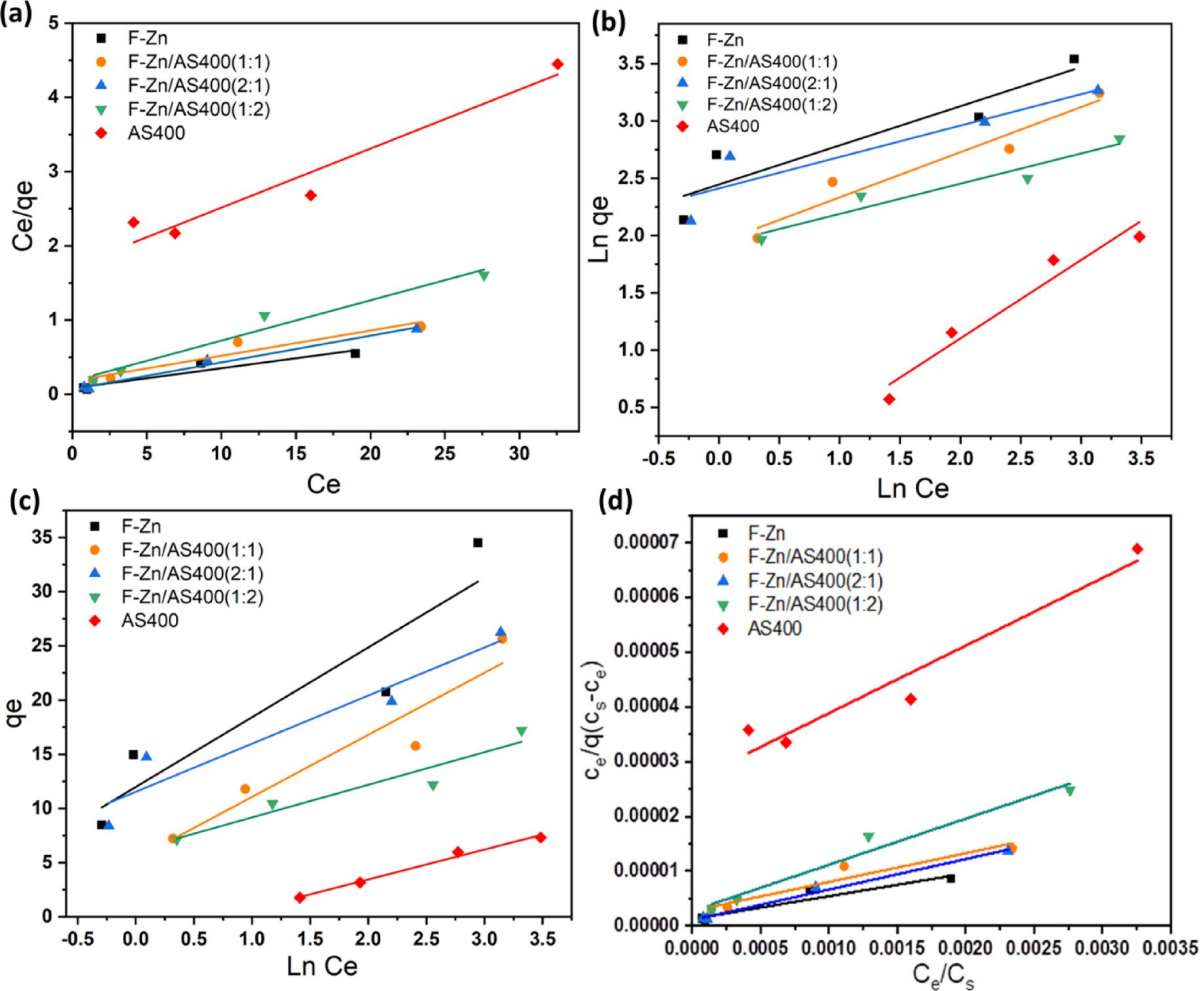
Adsorption isotherm models for IC removal (**a**) Langmuir, (**b**) Frendlich, (**c**) Temkin and (**d**) BET models.

Isotherm modeling is crucial since it gives information on the adsorbent’s capacity, or how much is needed to remove a unit mass of pollutant under the specified conditions. The adsorption isotherms that are most frequently used to describe the non-linear equilibrium of the adsorbate between the adsorbent and solution at a fixed temperature are the Freundlich, Langmuir, Temkin and Brunauer Emmett Teller isotherms. Adsorption and desorption rate equations are combined to create the Langmuir isotherm model, which can be expressed as follows^43,44^:

6 $$\frac{{{\text{C}}_{{\text{e}}} }}{{{\text{q}}_{{\text{e}}} }} = \frac{1}{{{\text{K}}_{{\text{L}}} }} + \frac{{{\text{a}}_{{\text{L}}} }}{{{\text{K}}_{{0{\text{L}}}} }}{\text{C}}_{{\text{e}}}$$

7 $${\text{q}}_{{\text{m}}} = \frac{{{\text{K}}_{{\text{L}}} }}{{{\text{a}}_{{\text{L}}} }}$$

8 $${\text{R}}_{{\text{L}}} = \frac{1}{{\left( {1 + {\text{K}}_{{\text{L}}} } \right)}}$$

where the Langmuir constants for adsorption energy and binding site affinity are denoted by K_L_ and a_L_. q_m_ is the monolayer adsorption capability. The equilibrium parameter or separation factor R_L_, which forecasts the suitability or unsuitability of the adsorption mechanism under investigation, can be used to explain the fundamental features of the Langmuir isotherm. The value of R_L_ indicates if adsorption is irreversible (R_L_ = 0), favorable (0 < R_L_ < 1), linear (R_L_ = 1), or unfavorable (R_L_ > 1)^45^. The experimental data is plotted according such model in Fig. 11a.

##### Freundlich isotherm

The Freundlich isotherm model was examined to characterize the heterogeneous adsorption surface. Freundlich isotherm also describes the multilayer adsorption process. The following is the empirical equation for the Freundlich isotherm:

9 $$\ln \,q_{e} = \ln \,K_{f} + \frac{1}{n}\ln \,C_{e}$$

where n is the dimensionless heterogeneous constant associated with the adsorption intensity, C_e_ (mg/L) is the equilibrium IC concentration, q_e_ (mg/g) is the equilibrium adsorption capacity, and K_F_ (mg^1–1/n^L^1/n^g^−1^) is the Freundlich constant associated with the adsorption capacity^44^. The experimental data is plotted in the linearized form of Freundlich isotherm model as seen Fig. 11b.

##### Temkin isotherm

This model describes the adsorption process through a layer that is reduced in coverage due to the interactions between the adsorbent and the adsorbate. The equation that follows could be used to express the linearized form:

10 $$q_{e} = {\text{B}}\,\ln \,A + B\,\ln \,C_{e}$$

where B is related to the heat of adsorption (B = $\frac{RT}{b}),$ T is the absolute temperature (K), R is a gas constant (8.314 J/ mol K), A is the binding constant at equilibrium (Fig. 11c).

Figure 10b show Freundlich’s graph for the removal of IC on F-Zn and their composites with AS400. When “n” is more than 1, it indicates that the adsorbate removal procedure is physical adsorption and favourable.

##### BET model

In 1938, Edward Teller, Paul Emmett, and Stephan Brunauer suggested an isotherm model that specifies such BET isotherm potential. The BET formula can be expressed according to the following relation:

11 $$\frac{{{\text{C}}_{{\text{e}}} }}{{{\text{q}}_{{\text{e}}} \left( {q_{e} - q_{e} } \right)}} = \frac{1}{{q_{m } C_{BET} }} + \frac{{({\text{C}}_{{\text{BET }}} - 1)}}{{q_{m } C_{BET} }} \frac{{{\text{C}}_{{\text{e}}} }}{{{\text{C}}_{{\text{s}}} }}$$

where C_BET_ represents the surface binding energy, q_m_ represent the monolayer adsorption capacity, C_e_ is an equilibrium concentration and C_s_ is a concentration when saturation is occurs^47^. Generally, BET isotherm model performs better wen the adsorption is signified as a physisorption, but, Langmuir isotherm model is often better for the chemisorption adsorption nature.

Figure 11d exhibited BET isotherm graph for the removal of IC on F-Zn and their composites with AS400. According to the slope and the intercept data, C_BET_ and q_m_ can be computed, respectively. Also, all the studied models’ parameters are tabulated in Table 3.

**Table 3:** Adsorption isotherms parameters for various studied adsorption systems.

Isotherm parameters	F-Zn	F-Zn/AS400 (1:1)	F-Zn/AS400	F-Zn/AS400	AS400
			(2:1)	(1:2)	
Langmuir					
q_m_ (mg/g)	37.17	27.76	29.42	18.41	12.57
a_L_, L/mg	0.33	0.19	0.52	0.3	0.05
K_L_	12.13	5.57	14.38	5.55	0.58
R^2^	0.92	0.92	0.99	0.97	0.94
Freundlich					
K_F_	6.65	5.27	6.55	5.23	0.72
n	2.93	2.54	3.65	3.79	1.46
R^2^	0.86	0.95	0.84	0.93	0.96
Temkin					
B (KJ/mol)	6.44	5.73	4.44	3.01	2.76
A (L/g)	5.06	2.54	7.07	5.58	2.03
R^2^	0.86	0.91	0.91	0.91	0.99
BET model					
q_m_ (mg/g)	241	179	190	119	80
C_BET_x10^3^	3.27	5.2	1.9	3.03	0.47
R^2^	0.92	0.99	0.92	0.97	0.94

The removal of IC dyes using the prepared adsorbents had corresponding n values from (1.46–3.79), indicating that the dyes’ elimination procedure is effective^7,46^. Overall, it appears that *R*^2^ value of the Langmuir and BET models were greater than that of the Freundlich and Temkin models, indicating that IC dye adsorption onto the F-Zn AS400 and their composites might follow Langmuir and BET isotherm model (Table 2). Such investigation correlates that IC molecules form a homogeneous monolayer and multilayer attributed the observed type II isotherms^46,47^. R_L_ are (0.0036- 0.0.021), (0.0037–0.025), (0.0039–0.026), (0.0037–0.021) and (0.004–0.029) for the F-Zn, F-Zn/AS400 (1:1), F-Zn/AS400 (1:2) and F-Zn/AS400 (2:1) respectively. Furthermore, adsorption is favorable according to the Langmuir model’s R_L_ values, which run from 0 to 1 and BET isotherm model^45^. Such investigation is in accordance with the previous work reported in literature that confirmed that the adsorption reaction is following Langmuir^48^ and BET model^49^., But, other work verifies the reaction is flowing Freundlich isotherm^50^.

#### Adsorption thermodynamics

**Fig. 12:**
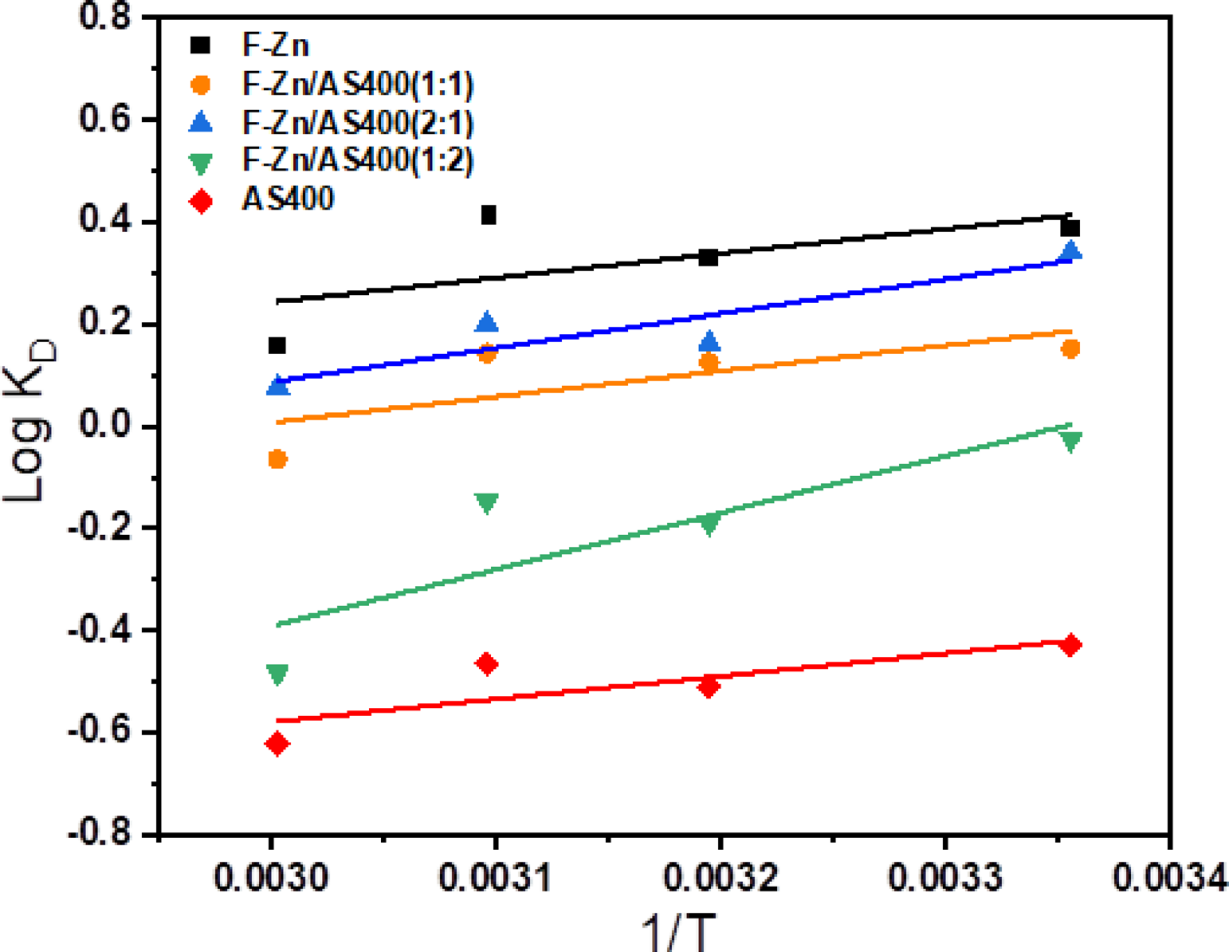
Van’t Hoff plot of Log K_D_ versus 1/T (K).

The spontaneity and suitability of the entire adsorption process were estimate thermodynamically by calculating the changes in Gibbs free energy (ΔG), enthalpy (ΔH), and entropy (ΔS) according to the following equations:

12 $${\text{K}}_{{\text{D}}} = \frac{{q_{e} }}{{C_{e} }}$$

13 $${\text{Log }}\,{\text{K}}_{{\text{D}}} = \frac{{{\mathbf{\Delta S}}}}{{\text{R}}} - \frac{{{\Delta H} }}{{2.303\,{\text{ RT}}}}$$

14 $$\Delta {\text{G }} = \, \Delta {\text{H }} - {\text{T }}\Delta {\text{S}}$$

where K_L_ is the equilibrium constant, q_e_ is the amount of dye adsorbed on the adsorbent per liter of solution at equilibrium (mg/l) and C_e_ is the equilibrium concentration in solution (mg/l). R is the universal gas constant (8.314 J/mol K) and T is the absolute temperature (K). ΔG (kJ/mol), ΔH (kJ/mol) and ΔS (J/K mol) indicate changes of Gibbs free energy, enthalpy and entropy, respectively. ΔH and ΔS could be calculated by plotting log K_D_ vs. 1/T from the slope, and the intercept, as shown in Fig. 12.

The resulting thermodynamic constants are detailed in Table 4. Negative ΔG values of F-Zn, F-Zn/AS400 (1:1) and F-Zn/AS400 (2:1) confirm the spontaneity of the indigo carmine dye removal process, while the other prepared adsorbents have positive ΔG value indicating that the adsorption is non-spontaneous^21^. The obtained ΔH values have negative charge indicating that the removal process was exothermic. The negative entropy could suggest a decrease in the degree of freedom.

**Table 4:** Thermodynamic parameters for dye adsorption on different adsorbents.

Adsorbent	T (K)	log K_D_	ΔG (K/mol)	ΔH (KJ/mol)	ΔS (J/mol K)
F-Zn	298	0.38	− 2.19	− 9.16	− 22.81
	313	0.33	− 1.97		
	323	0.41	− 2.55		
	333	0.16	− 0.99		
F-Zn/AS400(1:1)	298	0.15	− 0.87	− 9.64	− 28.77
	313	0.13	− 0.75		
	323	0.14	− 0.89		
	333	− 0.06	0.41		
F-Zn/AS400(2:1)	298	0.34	− 1.95	− 12.87	− 36.96
	313	0.16	− 0.98		
	323	0.2	− 1.24		
	333	0.08	− 0.49		
F-Zn/AS400(1:2)	298	− 0.03	0.14	− 21.24	− 71.21
	313	− 0.19	1.13		
	323	− 0.15	0.91		
	333	− 0.48	3.08		
AS400	298	− 0.43	2.44	− 8.58	− 36.81
	313	-0.51	3.05		
	323	-0.47	2.88		
	333	-0.62	3.96		

#### Comparative study of different adsorbents

To assess the present study performances, it is necessary to compare the data from the literature with the current work. Table 5 lists the IC removal capacity in comparison to various ferrite composites. Due to its microporous structure, the produced ferrite has the greatest BET surface area of any adsorbent. Additionally, it seems that the unhindered access of active sites, or oxygen anions, at the produced surface of ferrite nanoparticles contributed to the significant adsorption of F-Zn and their composites with AS400. Calcium and sodium aluminosilicate (Zeolite) are present, as shown by the X-ray diffraction data (Fig. 2). These elements are beneficial for the adsorption of dyes. The current investigation is also more environmentally friendly than the other recommended methods because ferrite and alum sludge are both safe for the environment.

**Table 5:** Adsorption capacity and time for adsorption removal of IC dye using various adsorbents.

Adsorbent*	Adsorption capacity (mg/g)	Operating time (min)	References
CBK1/1	18.29	80	^51^
NP-COM	6.12	150	^52^
NP-SYN	17.45	150	^52^
Zn-Cr-CO_3_	21.79	40	^53^
Hydrogel	3.57	1440	^54^
Ag_0.1_Zn_0.4_Ni_0.5_Fe_2_O_4_@mSiO_2_	33.1	110	^56,55^
MnFe_2_O_4_@MOAC	33.53	120	^56 ^
F-Zn	37.17	120	Current study
F-Zn/AS400 (1:1)	29.42	120	Current study

CBK1/1: activated carbons from Garcinia cola nut shells impregnated with KOH; NP-COM: commercial magnetite nanoparticles; NP-SYN: superparamagnetic magnetite nanoparticles; m: mesoporous.

## Conclusion

In current work, F-Zn and its novel composites augmented with modified alum sludge residual signified as AS400 and (F-Zn/AS400) in different proportions of (1:1), (1:2), (2:1) is introduced as a novel recyclable adsorbents. Furthermore, FTIR, XRD, SEM, TEM, EDX, BET, and VSM are applied to characterize the prepared adsorbents. Batch adsorption test is used an anionic dye (Indigo carmine IC) to check the materials’ adsorption tenancy. The system parameters are checked including pH, adsorbent dosage, initial dye concentration and temperature on the IC dye removal capacity. The removal was efficient at pH 2.0 using adsorbent dose of 0.5 g/L through 2 h of isotherm time. The adsorption capacity is improved from 12.57 to 29. 42 mg/g when F-Zn is added to AS400. Furthermore, Langmuir isotherm, which indicates monolayer coverage and the homogeneous nature of the IC dye adsorption on the adsorbents surface, was found to be the best fitting the experimental data. Kinetic models are also applied for practical applications and the data is well fitted with the pseudo-second order kinetic model. Also, thermodynamic data demonstrated that the adsorption process was exothermic in nature, whereas the reaction is ranged from spontaneous to non-spontaneous according to the adsorbent applied. Hence, according to the results of the current investigation, alum sludge waste is successfully introduced as a recyclable adsorbent in its composite form to well remove anionic dye from wastewater. Future studies might be required to examine these composites with other ratio in order to remediate more wastewater contaminants.

## Data Availability

The data that support the findings of this study are available from the corresponding author upon reasonable request.
